# Towards Portable Nanophotonic Sensors

**DOI:** 10.3390/s19071715

**Published:** 2019-04-10

**Authors:** Abdul Shakoor, James Grant, Marco Grande, David. R. S. Cumming

**Affiliations:** 1 Optoelectronics Research Centre, University of Southampton, Southampton SO17 1BJ, UK; 2 School of Engineering, University of Glasgow, Glasgow G12 8LT, UK; james.grant@glasgow.ac.uk (J.G.); david.cumming.2@glasgow.ac.uk (D.R.S.C.); 3 Dipartimento di Ingegneria Elettrica e dell’Informazione, Politecnico di Bari, 70125 Bari, Italy; marco.grande@poliba.it

**Keywords:** optical chemical sensors, nanostructured materials, refractive index, biosensors, lab on a chip

## Abstract

A range of nanophotonic sensors composed of different materials and device configurations have been developed over the past two decades. These sensors have achieved high performance in terms of sensitivity and detection limit. The size of onchip nanophotonic sensors is also small and they are regarded as a strong candidate to provide the next generation sensors for a range of applications including chemical and biosensing for point-of-care diagnostics. However, the apparatus used to perform measurements of nanophotonic sensor chips is bulky, expensive and requires experts to operate them. Thus, although integrated nanophotonic sensors have shown high performance and are compact themselves their practical applications are limited by the lack of a compact readout system required for their measurements. To achieve the aim of using nanophotonic sensors in daily life it is important to develop nanophotonic sensors which are not only themselves small, but their readout system is also portable, compact and easy to operate. Recognizing the need to develop compact readout systems for onchip nanophotonic sensors, different groups around the globe have started to put efforts in this direction. This review article discusses different works carried out to develop integrated nanophotonic sensors with compact readout systems, which are divided into two categories; onchip nanophotonic sensors with monolithically integrated readout and onchip nanophotonic sensors with separate but compact readout systems.

## 1. Introduction

The drive to improve healthcare has prompted researchers to develop high performance sensors to enable diagnosis of diseases at an early stage. On-chip nanophotonic sensors are an excellent candidate technology since they hold the potential to deliver high performance and compact sensors [1,2,3,4,5,6,7,8,9,10,11,12,13,14,15,16,17,18,19,20,21]. In particular, integrated nanophotonic optical sensors have shown great progress in achieving high sensitivity and low detection limit, with values as good as 1000 nm/refractive index unit (RIU) [22,23] and pg/mL [24,25] respectively, already reported. Nanophotonic sensors that are integrated on to a single chip are composed of different materials such as metals [26,27,28,29,30,31], semiconductors [32,33,34,35,36,37], dielectrics [38,39,40], or polymers [41,42], and consist of different device configurations such as an array of nanostructures [43,44,45,46], cavities [47,48,49,50,51,52], waveguides [53,54,55,56], interferometers [12,57,58,59], or gratings [60,61,62,63,64]. These sensors are mostly based on the phenomenon of refractive index sensing [65]. In these devices the signal output is altered by a change of the refractive index in close proximity to the sensor that is induced by applying an analyte. The output signal can be either a shift in the resonance wavelength for resonant based sensors [43,44,45,46,47,48,49,50,51,52] or a change in the transmission level for waveguide [53,54,55,56] or interferometric devices [12,57,58,59]. Mostly, these sensing devices require external bulky and expensive equipment such as external detectors or spectrum analyzers to measure the output signals, making their commercial application limited, especially where miniaturization would be an advantage. There are examples of commercial sensor systems that use nanophotonic structures, but their use is still limited by the large size of the equipment used for their readout. Notable examples are Biacore [66], which is based on surface plasmon resonance (SPR), and Genalyte [67], which uses ring resonators. Thus, although the nanophotonic sensing chips are themselves compact their readout requires a laboratory setup that makes the use of nanophotonic sensors in everyday life impractical. The need for large laboratory equipment gives rise to the so called “chip in a lab dilemma” for nanophotonic sensors. To transform nanophotonic sensors from laboratory-based demonstrations into practical devices that can be commercialized and used easily in everyday life, it is necessary to develop a compact nanophotonic sensor system, where not only the sensor chip itself, but its readout system is also compact and easy to operate by a non-expert. There have been several efforts to develop on-chip nanophotonic sensors with compact readout systems where researchers have taken different approaches [68,69,70,71,72,73,74,75,76,77,78]. In this article, we will give an overview of the work that has been done to date to develop on-chip nanophotonic sensors with compact readout systems. This article is organized as follows; first, the physical phenomena on which nanophotonic sensors operate along with associated performance parameters will be discussed; a brief summary of different types of on-chip nanophotonic sensors made using different materials such as metal (plasmonic), dielectric and semiconductor will then be provided; and finally, an overview of the efforts made so far to miniaturize the readout system of the nanophotonic sensors will be presented. In this review, the sensors having compact readout system are divided into two categories, namely: those where sensors have a monolithically integrated readout system [68,69,70,71,72,73,74,75,76] and those where the readout system is compact but separate to the sensor chip [77,78]. The commonest transducer to capture signals into the electronic domain is a semiconductor photodiode (PD). To develop nanophotonic sensors with a monolithically integrated readout system, it is increasingly commonplace to use a complementary metal oxide semiconductor (CMOS), photodiode device or array, and these will be discussed in this review.

## 2. On Chip Nanophotonic Sensors

**Figure 1:**
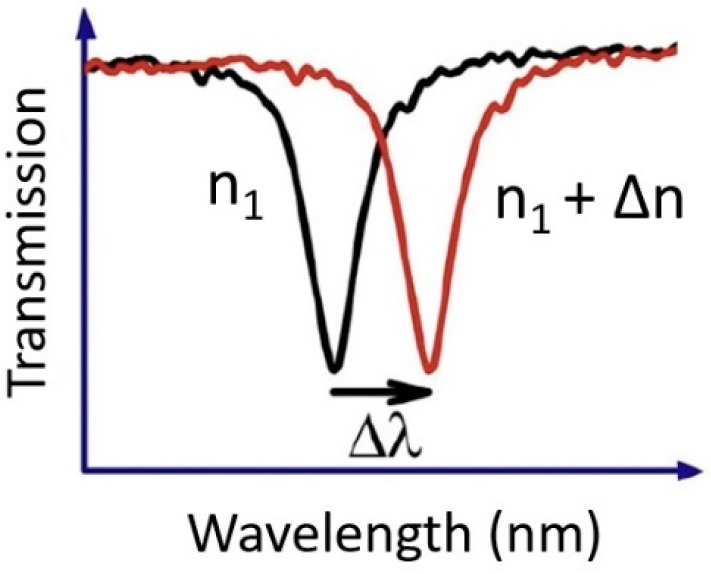
Resonance wavelength shift due to change in the refractive index. Reprinted with permission from [89]. Copyright © Elsevier (2012).

Label free sensing is increasingly important in the implementation of portable and ready-to-use optical sensors [79,80,81,82,83,84,85,86]. Refractive index sensing is therefore an ideal method since other established techniques, such as fluorescence sensing require a labelling technology to work [87,88]. In refractive index sensing the transmission/reflection signal from the nanophotonic structures changes by changing its localized or surrounding refractive index on introducing an analyte or binding a biological specie [65]. Most common nanophotonic refractive index sensors are made of resonant nanostructures [43,44,45,46,47,48,49,50,51,52], whose resonance wavelength shifts due to a change in the refractive index on applying any measurand such as a chemical or a biological specie, as shown in Figure 1. 

The measurand is quantified from the magnitude of the resonance shift. Apart from resonant nanophotonic sensors, there are other configurations available, including waveguide based sensors [53,54,55,56], where the absorption of an analyte causes a change in the transmission intensity and interferometric sensors, where a change in the localized refractive index of one arm of an interferometer, changes the output signal of the interferometer [12,57,58,59]. In this review, we shall focus on label free on-chip resonant refractive index sensors. The two main performance parameters of resonant nanophotonic refractive index sensors are sensitivity and detection limit [65]. The sensitivity is defined as a ratio of the magnitude of the resonance wavelength shift of the nanostructure to the induced refractive index change and is expressed in the units of nm/refractive index unit (RIU). The optical mode in the resonator decays evanescently into the surrounding analyte and the sensitivity depends on the degree of the modal overlap with the analyte. The larger the overlap, the greater the sensitivity. In addition to sensitivity, the detection limit is an important performance parameter for nanophotonic sensors and is defined as the capability of the sensor to measure the least possible quantity of the specie of interest. The value of the detection limit depends on the sensitivity of the sensor and the resolution of the system used for the measurements [65]. 

In the following section, a brief overview of different state-of-the-art resonant nanophotonic sensors composed of different materials such as metal, dielectric and semiconductor is presented.

### 2.1. Metal-Plasmonic Nanophotonic Sensors

**Figure 2:**
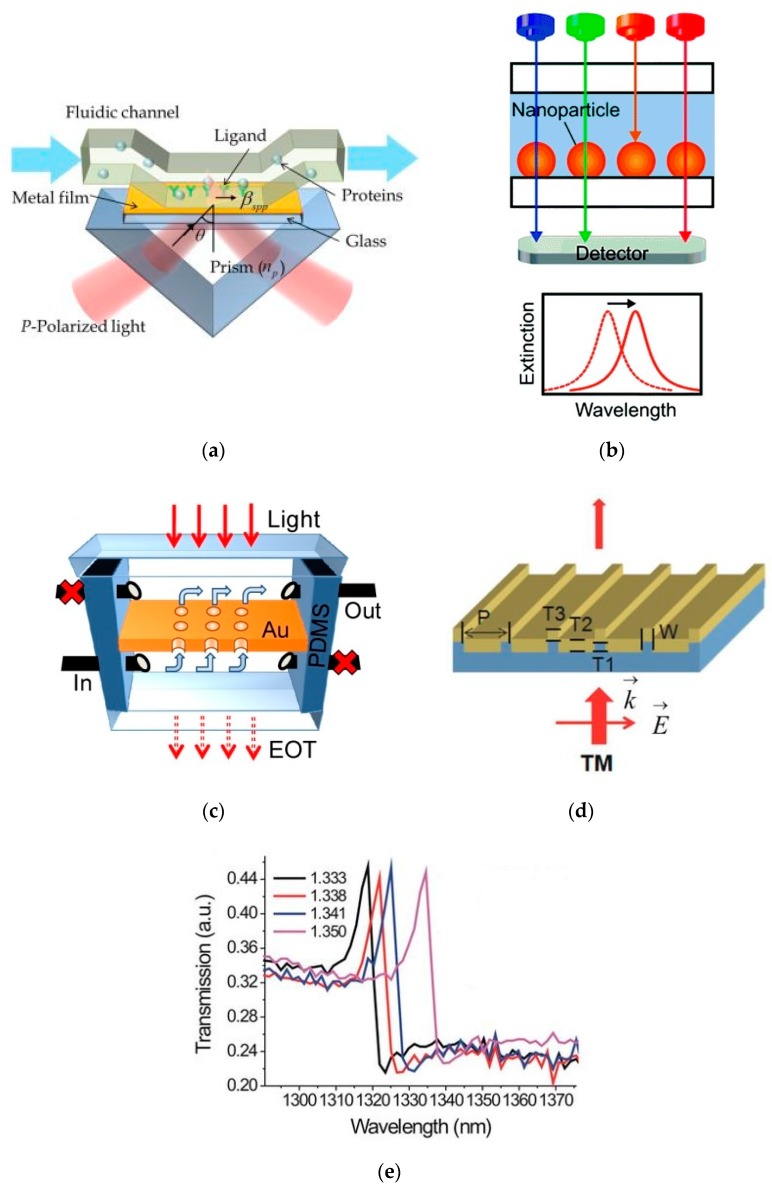
Schematic of plasmonic sensors. (**a**) Surface plasmon resonance (SPR) sensor requiring prism coupling. Reprinted from [93] under CC BY 3.0. (**b**) Localized surface plasmon resonance (LPSR) based sensor composed of an array of metallic nanoparticles. Reprinted with permission from [110] under CC BY-NC 3.0. Copyright © The Royal Society of Chemistry (RSC) (2014). (**c**) An array of metallic nanoholes operating on the principle of extraordinary optical transmission (EOT). Reprinted with permission from [111]. Copyright © SPIE (2010). (**d**) Schematic of plasmonic structures having Fano resonance. (**e**) Fano resonance shift by change of refractive indices. (**d**,**e**) Reprinted from [103] under CC BY 4.0.

Nanophotonic sensors made of metal exploit the phenomenon of surface plasmon resonance (SPR) [90,91,92,93]. Surface plasmon resonance is a resonant oscillation of the metal’s conduction electrons induced by light incident on its surface. Metal has a negative permittivity while its cladding such as air has positive permittivity. The conduction electrons resonance induced by the incident light generates plasmonic modes at the interface of the negative (metal) and positive (dielectric) permittivity material, which can travel across the interface. The plasmon resonance causes enhancement of the electric field at the interface of the metal and dielectric. When a biological species is bound to the metal, it interacts with the high electric field intensity at the interface resulting in high sensitivity values. However, SPR sensors require a prism-coupling scheme, which adds to additional complications in the measuring setup. If the continuity of the metal is broken, such as in a nanoparticle, the plasmon mode is localized at the interface with the air and is called localized surface plasmon resonance (LPSR), which is exploited to develop high sensitivity sensors [94,95,96,97,98]. Another phenomenon called extraordinary optical transmission (EOT) through an array of metallic holes is utilized to make high performance optical sensors [4,5,31,43]. Plasmonic sensors based on metallic nanostructures that give a Fano resonance response are also reported [99,100,101,102,103,104]. Fano resonance is a type of resonance which has an asymmetric line shape and narrower linewidth compared to the standard Lorentzian resonance and has the potential to develop high sensitivity plasmonic sensors. Fabricating an array of metal nanostructures such as nanodiscs, nanoparticles or nanoholes generates an array of electric field hotspots giving rise to a resonant transmission/reflection behavior, which is utilized for sensing. The measurement of LSPR, EOT, or Fano resonance does not require prism coupling which simplifies the measurement setup to some extent. However, these techniques still need an external spectrometer or detector to record the resonant transmission/reflection response. Nanophotonic sensors exploiting propagating [90,91,92,93] and localized SPR [94,95,96,97,98], EOT [4,5,31,43], and Fano resonance [99,100,101,102,103,104] are reported, examples of which are provided in Figure 2. Plasmonic nanophotonic sensors can achieve very high sensitivity values (few hundreds of nm/RIU) [43,98,99,100,101,102,103,104,105,106,107,108,109,110,111] and even higher (thousands of nm/RIU) by metamaterial designs [23]. However, their readout requires external bulky equipment, limiting their use to laboratory environments.

### 2.2. On Chip Semiconductor/Dielectric Nanophotonic Sensors

**Figure 3:**
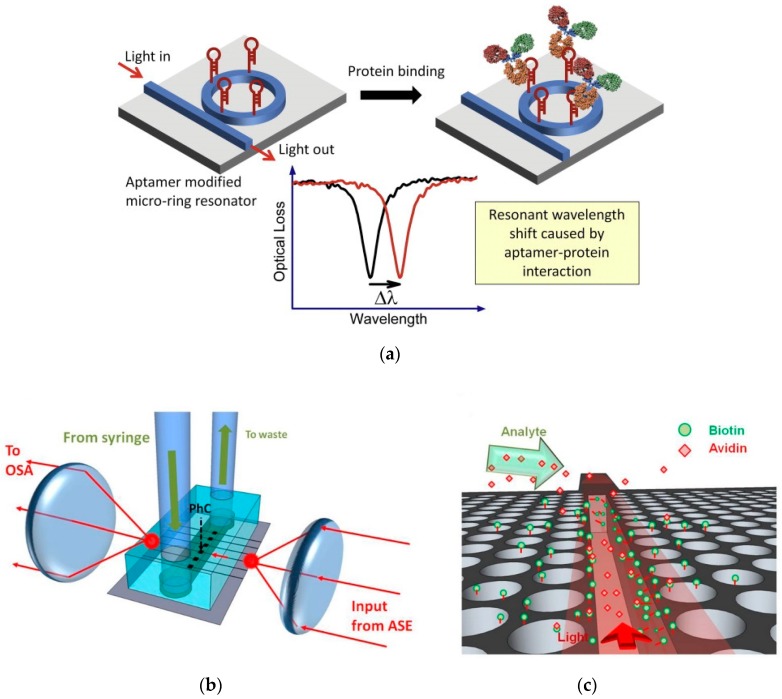
Schematic of semiconductor cavity-based sensors. (**a**) Ring resonator sensor. Reprinted with permission from [89]. Copyright © Elsevier (2012). (**b**,**c**) Slotted photonic crystal cavity sensor. Reprinted from [118] under CC BY 3.0. Both types of sensors require external measuring equipment such as optical spectrum analyzers (OSA) to measure the small magnitudes of the wavelength shift.

Nanocavities made of semiconductor or dielectric materials such as silicon and silicon nitride respectively, can achieve very narrow resonance linewidth, i.e., in the order of picometers [18,112]. This very narrow resonance response is exploited to develop high performance sensors. The most notable onchip semiconductor cavity sensors are based on ring resonators [19,35,47], disk resonators [48,49] and photonic crystal cavities [113,114,115,116,117]. A special type of cavity in which the optical mode is confined in the air slot such as slotted photonic crystal cavity [18,112,118,119] and slotted ring resonator [17,120,121] is developed to enhance the overlap of the optical mode and the analyte to increase the sensitivity. In addition to high Q-factor cavities, grating designs having narrow transmission/reflection response are utilized for sensing [60,61,62,63,64,122]. Nanocavities or gratings made of silicon operate at sub-bandgap wavelengths (wavelengths larger than 1100 nm) hence to perform sensing experiments in the visible range, nanocavities or gratings made of silicon nitride are used [17,38,64,123]. The main advantage of using semiconductor/dielectric nanocavities and gratings for sensing is achieving very high detection limit. However, to measure the resonance wavelength shift which is in the orders of nano or picometers, costly spectrometers are needed which makes the sensors expensive and impractical. Schematic examples of high-performance cavity based sensors is shown in the Figure 3.

## 3. Nanophotonic Sensors with Compact Readout System

This section provides an overview of different efforts done to develop nanophotonic sensors with compact readout system. These works can be divided into two main categories; (i) nanophotonic sensors having monolithically integrated readout system and (ii) nanophotonic sensors having compact but separate readout system.

### 3.1. Nanophotonic Sensors having Monolithically Integrated Readout System

**Figure 4:**
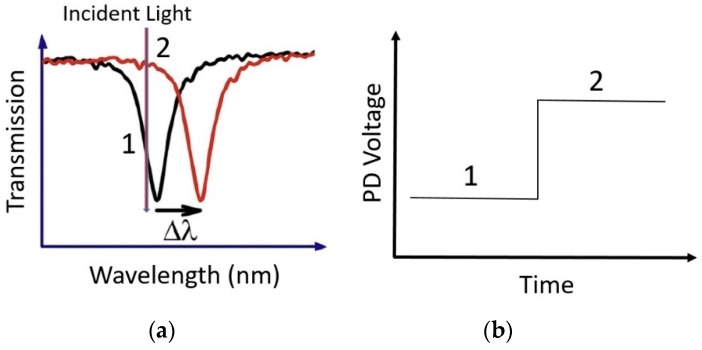
(**a**) Resonance wavelength shift due to change in the refractive index. Keeping the incident wavelength fixed (red vertical arrow), the intensity of light impinging on the PD changes from level 1 to level 2 resulting in the increase in the PD output voltage/current from level 1 to 2 as shown in (**b**).

One approach to developing a portable nanophotonic sensor having a compact readout system is to integrate nanophotonics structures monolithically with the photodiodes (PDs) having high responsivity in the region of interest. By doing so, the resonance wavelength shift can be measured as a change in the electrical output (current, voltage) of the PD as shown in the Figure 4. 

In contrast to standalone nanophotonic sensors where performance is usually quoted in terms of the resonance wavelength shift in response of unit refractive index change, in the integrated system the important parameter is not merely resonance wavelength shift but the change in the optical intensity impinging on the PD due to the wavelength shift. There are three parameters that control the change in the optical intensity impinging on the PD due to wavelength shift, i.e. sensitivity, resonance linewidth and resonance depth, which is defined as the difference between the transmission/reflection level of the resonance dip/peak and the background level. The higher the sensitivity, greater will be the resonance shift per unit refractive index change causing a larger change in the intensity. Similarly, the narrower the linewidth, the greater will be the intensity change for certain wavelength shift provided the linewidth of the incident light is smaller than the resonance response of the nanostructures. In the same way, the greater the resonance depth, the larger will be the intensity change induced by the wavelength shift. In integrating nanophotonic structures with PDs, it is important to design and optimize the nanophotonic structure in order to maximize the intensity change for a certain wavelength shift. Furthermore, the choice of nanophotonic structures for integrating with the PD depends on the applications and requirements of the sensors. Plasmonic structures offer high sensitivity however due to high optical losses, have broad resonance linewidths. On the other hand, semiconductor or dielectric structures such as cavities and gratings have moderate sensitivities compared to plasmonic structures however they have narrower linewidths and a greater resonance depth, resulting in a greater change in the intensity impinging on the PD due to resonance wavelength shift.

#### 3.1.1. Nanophotonic Sensors with Integrated Silicon Photodiode

**Figure 5:**
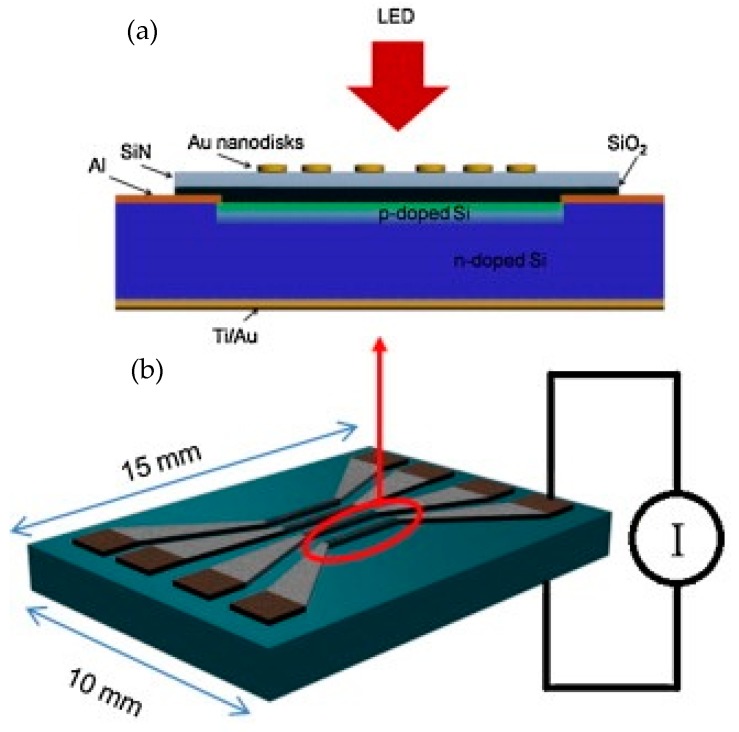
Schematic of the device detailed in Mazzotta et al. [68]. (**a**) Cross section of the photodiode (PD) with integrated gold nanodiscs. (**b**) Schematic of the sensor chip having four PDs with dimensions of 2 mm × 0.14 mm. Two PDs had gold nanodiscs fabricated on top while the other two PDs were used as a reference. The total size of the sensor chip is 15 mm × 10 mm. Reprinted with permission from [68]. Copyright © Elsevier (2010).

An initial effort in developing nanophotonic sensors with compact readout system was presented by Mazzotta et al. [68]. In this work, nano plasmonic structures (gold nanodisc array) were monolithically integrated with the bulk silicon photodiode (PD), as shown in Figure 5. The best results were obtained when the thickness and diameter of the gold nanodiscs were 20 nm and 110 nm respectively. These parameters gave a resonance at 650 nm, measured by fabricating the gold nanodisc array on a test silicon nitride coated glass slide. The optical sensitivity response was measured by applying different concentrations of glycerol as an analyte on the gold nanodisc array fabricated on the silicon nitride coated glass slide.

The resonance wavelength of the gold nanodisc array red-shifted by applying different concentrations of glycerol. From the redshift of the resonance wavelength, the optical sensitivity value of the gold nanodisc array was measured to be 133 nm/RIU, which is a typical sensitivity value for these types of plasmonic structures. The gold nanodisc array having similar dimensions as in the test sample was fabricated on top of silicon nitride coated bulk silicon photodiode (PD). The top silicon area was p-doped while rest of the bulk substrate was n-doped to create top and bottom metal contacts, respectively. By monolithically integrating the gold nanodisc array with the silicon PD, its optical transmission response was converted to an electrical signal and hence the need for external equipment to measure the optical response was eliminated. When the resonance wavelength of the gold nanodisc array red shifted due to applying an analyte, such as glycerol, the optical intensity incident on the monolithically integrated PD changed which in turn changed the PD output current. 

**Figure 6:**
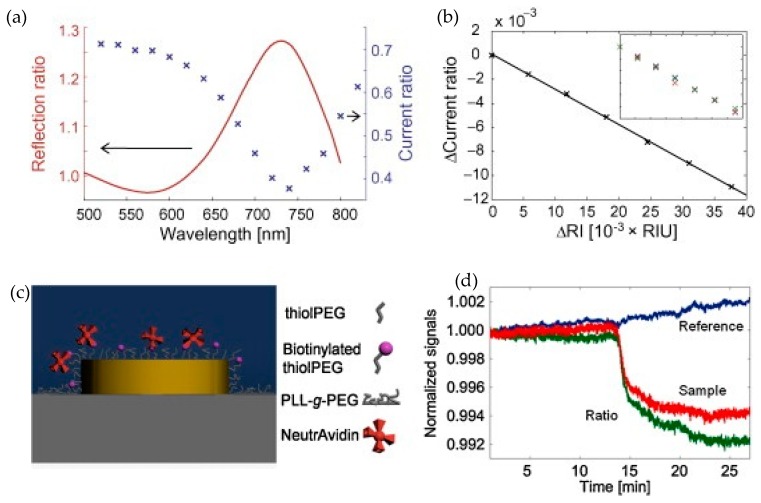
Measurement results of the sensor chip detailed in Mazzotta et al. [68]. (**a**) The red curve is the ratio between the reflection from the nanodiscs and the reference PD showing the reflection response of the gold nanodisc array. The blue curve represents the PD photocurrent ratio for different wavelengths of the incident light. (**b**) The graph represents the change in the PD current ratio versus change in the refractive while keeping the incident light fixed at 780 nm. (**c**) Graphical illustration of specific binding of neutravidin with gold nanodiscs. (**d**) Normalized photocurrent from reference PD (blue), PD with nanodisc array (red) and the photocurrent ratio (green). The curves are normalized with their initial values which were 1.679 V for the reference (blue) curve, 0.693 V for the PD with nanodiscs (red) and 0.413 V for the photocurrent ratio (green). Reprinted with permission from [68]. Copyright © Elsevier (2010).

The electrical sensitivity data was presented in terms of PD output current ratio, as shown in Figure 6, which is defined as the PD output current when analyte is applied versus the PD output current with air as the cladding (reference diode). From the PD output current ratio, the photocurrent ratio sensitivity, which is defined as the ratio of the change in the photocurrent ratio when analyte is applied to the corresponding change in the refractive index was measured to be 0.01/RIU.

From the photocurrent ratio, the sensitivity in terms of change in transmission or optical intensity was estimated to be −29%/RIU, which is less than a half compared to the change in transmission measured on a test glass slide sample (−70% RIU). The negative sign indicates decrease in transmission. The difference was attributed to the possible variation in plasmonic properties of the gold nanodisc array fabricated on the glass substrate and on the silicon PD and also to the difference in the measurement setups. The capability of the integrated nanophotonic sensor to measure the specific binding of protein to receptors immobilized on the gold nanodisc surface was also shown by the change in the PD output signal as presented in the Figure 6d. 

**Figure 7:**
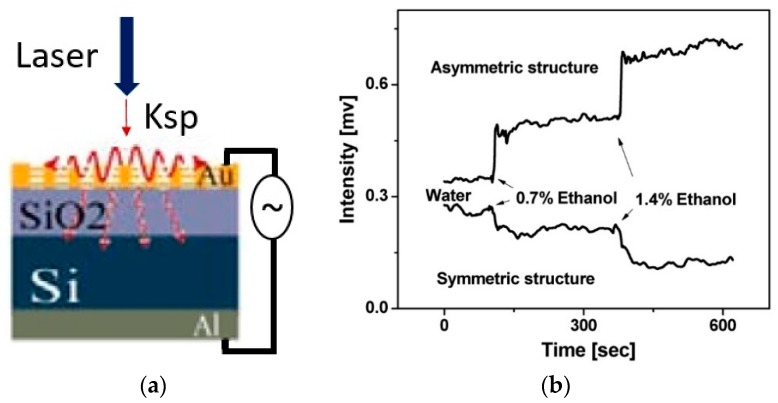
(**a**) Schematic of plasmonic nanostructures integrated with the silicon photodetector. (**b**) Output of the sensor showing measurements for symmetric and asymmetric plasmonic nanohole arrays when water and different concentrations of ethanol were applied. Reprinted with permission from [69]. Copyright © The Optical Society (OSA) (2011).

A similar approach was taken by Guyot et al. [69], where nanoplasmonic structures (array of gold nanoholes) were monolithically integrated with p-doped silicon substrate that acted as a photodetector in the metal oxide semiconductor (MOS) configuration. The array of nanoholes acted as a top contact while aluminum was deposited as a bottom metal contact, as shown in the Figure 7a. Following the same principle as explained in Section 3.1, the wavelength shift due to change in the refractive index was recorded as the change in the intensity detected by the photodetector. In this work, it was shown that the sensing performance is better when asymmetric nanohole arrays having different periods in the orthogonal directions [124] are used compared to the symmetric array, as shown in the Figure 7b. However, better sensing performance for the asymmetric nanohole array is obtained by measuring the signal difference between orthogonal polarizations and a self-referencing system to eliminate experimental noise. The system requires a complicated setup and hence negates the usefulness of the portable readout system. The sensing performance of the device was presented by measuring the detector signal shift when water and different concentrations of ethanol are applied to the device. From Figure 7b, it can be seen that the 0.7% ethanol in water, which corresponds to 1.75 × 10^−4^ RIU change induced 0.07 mV and 0.15 mV signal shift for symmetric and asymmetric nanoholes array, leading to the electrical sensitivity of 0.4 V/RIU and 0.8 V/RIU, respectively. 

The same group also integrated angle sensitive metallic nano gratings with the MOS silicon photodetector and performed angular scanning of the incident light [70]. The integrated sensor was able to distinguish between nitrogen and argon gases whose refractive index difference is 1.5 × 10^−5^.

Angle sensitive plasmonic nano gratings were also integrated with the bulk silicon photodetector in [71,72]. In [71], Perino et al. measured the sensitivity in terms of the change in the transmission intensity of the zero-order diffracted ray of the functionalized and unfunctionalized grating and reported its value as 2.94/RIU, while the detection limit of the integrated sensor was reported to be 2.2 × 10^−4^ RIU. 

In [72], Turker et al., deposited a thin silver layer on top of polymer gratings fabricated on top of the silicon photodetector to enable plasmonic coupling. The electrical sensitivity of the gratings integrated silicon photodetector sensor reported in [72] was 0.6 mA/RIU.

#### 3.1.2. Nanophotonic Sensor with Integrated Germanium Pin Photodiode

In order to develop a nanophotonic sensor with compact readout system, Augel et al. [73], fabricated plasmonic nanohole arrays by etching nanoholes directly in the top aluminum metal contact of the vertical germanium pin photodiode (PD). Germanium PDs were chosen instead of silicon because germanium has a higher absorption coefficient and offers a larger value of the generated photocurrent. Furthermore, the operation spectrum of germanium extends to the near infrared region and hence allows to perform sensing experiments in the near infrared range in addition to the visible wavelengths. 

**Figure 8:**
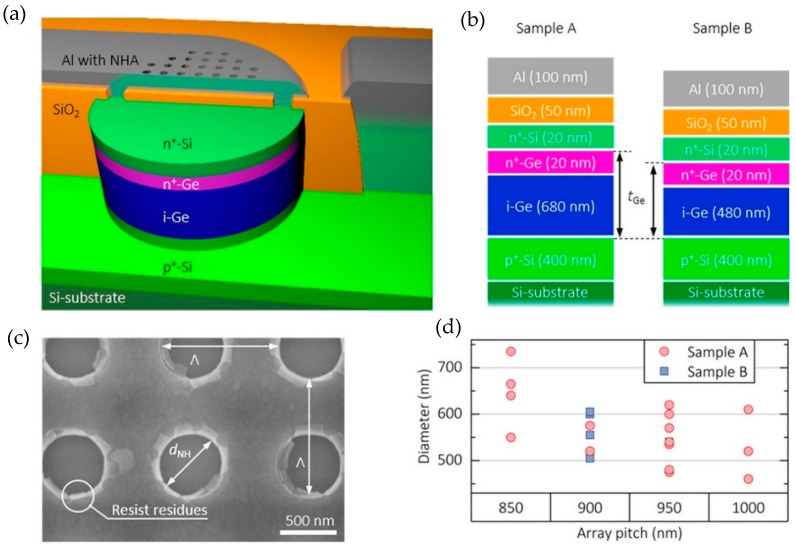
(**a**) Schematic illustration of the device presented in Augel et al. [73], showing the cross section of the germanium PD and integrated aluminum nanohole array in the metal contact area. (**b**) Schematic showing the layers of the germanium PD where the germanium layer thickness is 780 nm and 500 nm for sample A and B respectively. (**c**) SEM image of the aluminum nanohole array integrated with the germanium PD. (**d**) The physical parameters (hole diameter and the pitch) of various nanohole arrays fabricated in the metal contact area of the germanium PD. Reprinted with permission from [73]. Copyright © American Chemical Society (ACS) (2018).

The performance of the integrated sensing system was optimized by varying the germanium layer thickness to tune the responsivity of the detector and by controlling the resonance response of the nanohole array by optimizing the diameter and periodicity of the nanoholes. The nanohole array was designed to exhibit asymmetric Fano resonances that have narrower linewidths compared to the Lorentzian resonance response and promise higher sensitivity and lower detection limit. The schematic of the device along with the cross section of the germanium pin PD and the parameters of the plasmonic nanohole array is shown in Figure 8.

The readout of the integrated sensing system was given in terms of the responsivity spectrum of the germanium PD. The light transmitted from the nanohole array was incident on the monolithically integrated germanium PD and its responsivity spectrum was measured. As can be seen from the Figure 8, the responsivity spectrum of the germanium PD without the integrated nanohole array is flat while a Fano resonance peak is visible in the responsivity spectrum of the plasmonic nanoholes integrated PD. 

**Figure 9:**
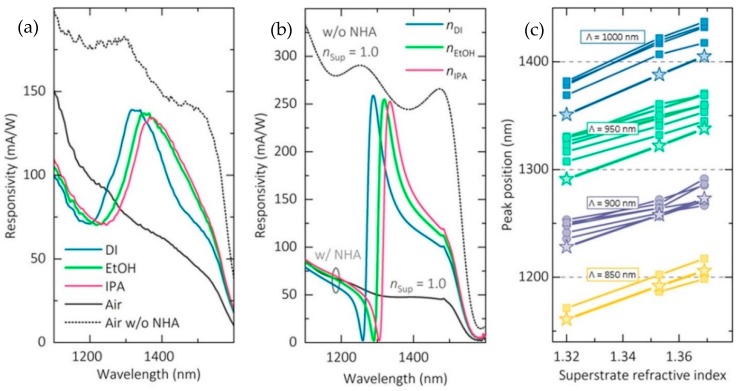
Experimental results detailed in Augel et al. [73]. (**a**) Experimental responsivity spectrum of the device for different refractive indices of the surrounding medium. The physical parameters of the nanohole array were as follows; pitch 950 nm, hole diameter 480 nm, germanium layer thickness 700 nm. (**b**) Simulation results for the responsivity of the device having parameters similar to (a). The peak position of the responsivity red shifts as the refractive index increases. (**c**) Responsivity peak positions versus refractive indices for different combinations of the device physical parameters. Reproduced with permission from [73]. Copyright © American Chemical Society (ACS) (2018).

The performance of the integrated sensing system was demonstrated by changing the refractive index of the surroundings of the nanohole array by adding DI water (n = 1.320), ethanol (n = 1.353), and isopropyl alcohol (IPA) (n = 1.369). As the refractive index of the solution increased, the Fano resonance of the nanohole array redshifted, which was read out as the redshift in the responsivity spectrum of the integrated germanium PD, as shown in Figure 9. From the measurements, the maximum sensitivity for the most optimized parameters of the device estimated from the germanium PD responsivity data was 1180 nm/RIU, which is even higher than the non-integrated plasmonic sensors.

#### 3.1.3. Nanophotonic Sensor Integrated with CMOS Technology

Complementary Metal Oxide Semiconductor (CMOS) technology has revolutionized the electronics industry especially in telecommunications and computing. The way CMOS technology has enabled widespread applications of electronic devices, it also holds promise to commercialize nanophotonic sensors if it can be integrated with the CMOS technology [125,126]. Integrating nanophotonic sensors with CMOS electronic technology combines the benefits of both nanophotonic sensors (high sensitivity and low detection limit) and the benefits of CMOS electronics such as compactness and ease of commercialization, thanks to the mature fabrication production lines of CMOS foundries. Keeping this in view, Shakoor et al. [74,75,76], performed the first monolithic integration of nanophotonic sensors with the photodiodes (PD) made by a CMOS foundry.

The CMOS PD integrated circuit was fabricated in a commercial CMOS foundry, namely Austria Microsystems using a 0.35 µm four metal layer high voltage CMOS technology. The dimensions of the individual PDs were only 6 × 8 µm which is much smaller compared to the millimeter scale PDs used in the works presented in Section 3.1.1 and Section 3.1.2. Miniaturized PDs is one of the main advantages of using detectors made by CMOS technology. Depending on the applications of interest, nanophotonic sensors made of different materials and designs can be fabricated on top of the CMOS PDs. In this work, plasmonic sensors composed of gold nano discs and dielectric sensors made of silicon nitride gratings were integrated with the CMOS PDs. The nanophotonic structures were optimized in terms of its resonance line width, resonance depth and operating range which should match the maximum responsivity of the PD to make it suitable for integration with the CMOS PDs. The plasmonic sensors offer greater sensitivity compared to the dielectric sensors, as discussed in Section 2, however due to the high losses associated with the metal, the resonance linewidth of the plasmonic structures is broad which reduces the magnitude of the intensity change induced by the resonance shift when an analyte is applied to the sensor at a fixed input wavelength. On the other hand, nanostructures exhibiting narrow resonance linewidths have limited dynamic range as the output signal saturates with just a small resonance shift. Thus, for applications where it is required to measure large changes in refractive index, nanostructures having broad resonance linewidths are appropriate, while on the other hand for measuring small changes in refractive index narrow band resonant structures such as gratings or cavities are a better option. In this work, both broadband plasmonic and narrow band dielectric structures were integrated with the CMOS PDs to demonstrate the potential of the integration approach.

**Figure 10:**
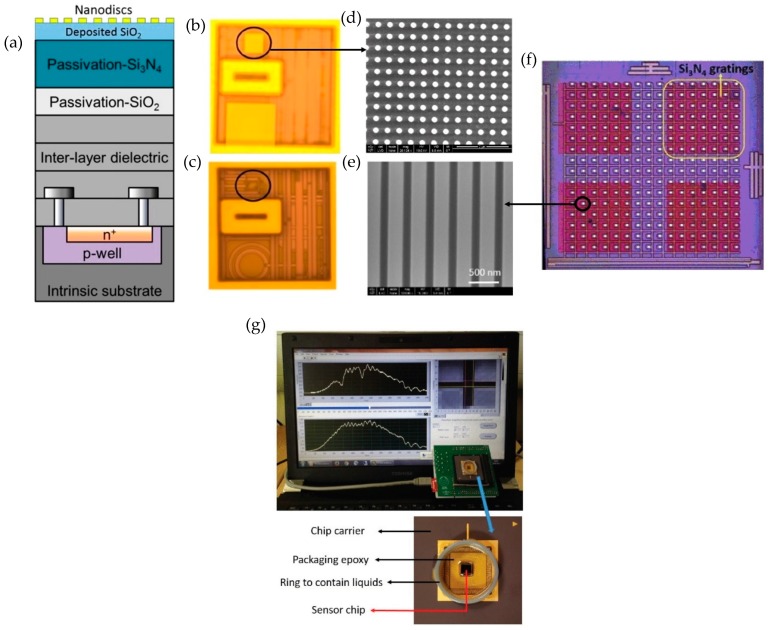
(**a**) Cross section schematic of the complementary metal oxide semiconductor (CMOS) PD chip with integrated gold nanodisc array. (**b**,**c**) Microscopic images of the CMOS PD chip. (**b**) with and (**c**) without integrated gold nanodisc arrays. The PD area is encircled in both cases and have dimensions of only 6 × 8 µm. (**d**) SEM image of the gold nanodiscs integrated on top of the CMOS PD chip. (**e**) SEM image of the silicon nitride gratings fabricated on top of the CMOS PD chip. (**f**) Microscopic image of the silicon nitride gratings integrated with the CMOS PD chip having an array of PDs. The four dark areas, one of which is encircled shows silicon nitride gratings. The PD pixels between the silicon nitride gratings were used as a reference. (**g**) Packaged sensor chip connected with the laptop with readout displayed on the laptop screen. (**a**–**d**) reprinted with permission from [74]. Copyright © American Chemical Society (ACS) (2016). (**e**–**g**) reprinted with permission from [76]. Copyright © IEEE (2018).

To integrate the nanophotonic sensors with the CMOS PDs, first a 500 nm thick layer of silica was deposited on top of the CMOS chip. The main reason for depositing this layer was to replicate the nanophotonic design optimized on a test glass slide sample. As the composition of the top passivation layer of the CMOS chip is not fully known, using the same design for the nanophotonic sensors as used in the test sample fabricated on the glass substrate could shift the resonance response if silica layer was not deposited on top of the CMOS chip. The nanophotonic structures were fabricated on top of the CMOS chip by standard e-beam lithography followed by lift off and etching processes for gold nanodiscs and silicon nitride gratings, respectively. The microscopic images of the gold nanodiscs and silicon nitride gratings fabricated monolithically on top of the CMOS chip along with the cross-section schematic of the CMOS chip are given in Figure 10.

The total size of the chip was only 3.5 × 4.5 mm. The nanophotonic sensor integrated CMOS chip was placed on a PGA-120 chip carrier, wire bonded to provide electrical connections and packaged by using the EPO-TEK 302 epoxy to allow sensing experiments with aqueous solutions. A printed circuit board controlled by a Labview program was used to power the chip. The Labview program read the real time voltage signal from the PD in a synchronous manner. The electrical data from the sensor system was recorded by computer by connecting it to the PCB via a USB cable. The system can also be easily connected wirelessly to a smartphone or a tablet. A photograph of the integrated sensor placed on a chip carrier and PCB is shown in Figure 10g.

**Figure 11:**
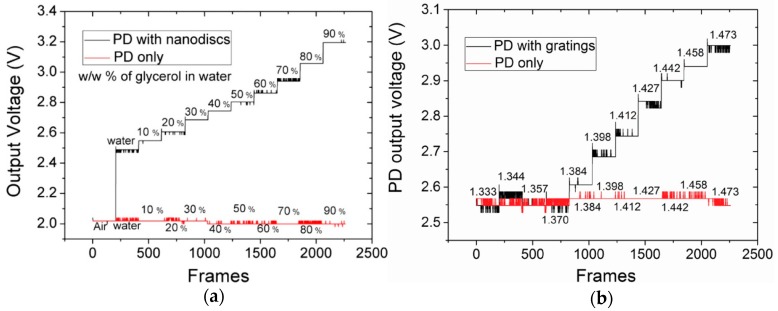
Measured changes in the PD output voltage for various glycerol concentrations as detailed in Shakoor et al. [74,76]. The black staircase curve is the readout from the PD with integrated nanophotonic sensors while the flat red curve corresponds to the readout from the PD with no nanophotonic structures. (**a**) Measured PD voltage for the gold nanodisc array sensor. (**b**) Measured PD voltage from the silicon nitride grating sensor. In (a) the data for the PD output voltage is presented for different concentrations of glycerol while in (b) the data is presented in terms of the refractive indices of different concentrations of glycerol. (**a**) reprinted with permission from [74]. Copyright © American Chemical Society (ACS) (2016). (**b**) reprinted with permission from [76]. Copyright © IEEE (2018).

The performance of the sensor system was evaluated by changing the refractive index of the surroundings by introducing different concentrations of glycerol. As the glycerol concentration was increased, the resonance peak red-shifted. The optical sensitivity values measured from test samples for gold nanodisc arrays and silicon nitride gratings were 275 nm/RIU and 160 nm/RIU, respectively. Keeping the wavelength of the input light fixed, the resonance-shift due to the change in refractive index changed the intensity of light impinging the underlying active layer of the PD, which in turn resulted in an increase or decrease of the PD output voltage. The change in the PD output voltage when different concentrations of glycerol were applied on top of the gold nanodiscs and silicon nitride gratings is shown in Figure 11. For the gold nanodisc integrated sensor, the change in PD output voltage with change in refractive index is linear. On the other hand, for the silicon nitride grating sensor the PD output voltage is not linear. This is because the resonance linewidth of the silicon nitride grating is narrow, and the magnitude of the resonance shift for different concentrations of glycerol depends on the chosen incidence wavelength. From the curve shown in Figure 11, the electrical sensitivity of the CMOS PD integrated gold nanodisc array sensor was estimated to 5.6 V/RIU, while the maximum sensitivity for silicon nitride grating was measured to be 6.75 V/RIU. The quoted electrical sensitivity values depend highly on the design of the electronic readout circuitry and hence can vary among different electronic readout systems. The estimated detection limit of the integrated detection system was 0.003 RIU, which is lower compared to standalone resonant nanophotonic sensors measured optically by high resolution spectrometers such as ring resonators. The main reason for lower detection limit is that the nanophotonic sensors are integrated with the CMOS chip which has a thick passivation layer and many layers of interlayer dielectrics. These layers can cause signal loss by back reflections which compromises the performance of the device. The performance of the device can be improved by removing the passivation layer and by carrying out improvements in the readout circuitry to reduce the signal to noise ratio. In addition to glycerol, as a proof of concept, the electrical readout of 1 mg/mL protein deposited on the gold nanodiscs was also shown in this work. Although the nanophotonic sensors were integrated with the CMOS PD chip by post processing using electron beam lithography in a non-CMOS clean room, it is a major step forward in developing a compact handheld nanophotonic sensor system having an integrated readout system.

To enable real commercialization the concept can be extended to fabricate the nanophotonic sensors in the CMOS pilot line instead of post processing. Mass production in a CMOS foundry would reduce the cost considerably.

### 3.2. Nanophotonic Sensors with Compact but Separate Readout System

Another approach adopted by researchers to develop sensors with compact readout system is to use imagers such as CMOS or Charge Coupled Device (CCD) imagers. Following this approach, the need of expensive, bulky and external equipment such as spectrometers is eliminated. However, in these approaches the sensor chips and the imagers are separate and need housing and alignment. In the following sections, an overview of different efforts carried out to develop nanophotonic sensors with compact readout system by using compact imagers is given.

#### 3.2.1. Nanophotonic Sensor Based on CMOS/CCD Imager for Readout

**Figure 12:**
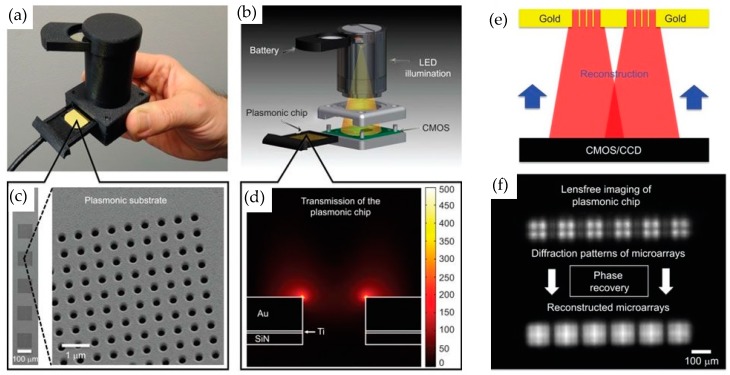
(**a**) Picture of the developed sensing device with sensor and imaging chip placed in compact housing as presented in Cetin et al. [77]. The total height of the device is only 7.5 cm and weighs only 60 g. (**b**) Schematic showing the components of the sensor system. (**c**) SEM image of the metallic nanohole array. (**d**) Simulation of the plasmon resonance showing intense electric field at the edges of the holes. (**e**) Illustration of the numerical technique for the reconstruction of the diffraction patterns of the nanohole array by using the phase retrieval method. (**f**) Recorded diffraction patterns of six metallic nanohole arrays (top line) and the reconstructed diffraction images by the phase retrieval method (bottom line). The distance between the nanohole array and the imager is 2 mm and no lens was used to focus the patterns. Reprinted with permission from [77]. Copyright © CIOMP (2014).

To get rid of the bulky external equipment to measure nanophotonic sensor chips, Cetin et al. used a lens free computational imaging system to record the transmission response of the plasmonic nanostructures [77]. In this work, CMOS/CCD imaging sensor was used to record the changes in the diffraction patterns of the plasmonic nanohole array due to the optical resonance wavelength shift. Following this approach, a handheld nanophotonic sensing system having 7.5 cm height and 60 g weight was demonstrated. The photograph of the packaged sensor developed in this work is shown in Figure 12a. The complete sensor system consists of a battery, LED light source, plasmonic chip consisting of an array of nanoholes and an imager. The battery, LED and the imager are enclosed in a housing while the plasmonic sensor chip is slid into the housing as shown in Figure 12b. An LED is used as an excitation source while the diffraction patterns generated by the plasmonic modes of the nanoholes array are imaged by the CMOS/CCD imaging chip which can be bought off the shelf. To enable multiplexed measurements, an iterative phase retrieval-based image reconstruction method was also employed to reconstruct the overlapped diffraction patterns from simultaneous measurements. The reconstructed diffraction patterns are shown in Figure 12f.

**Figure 13:**
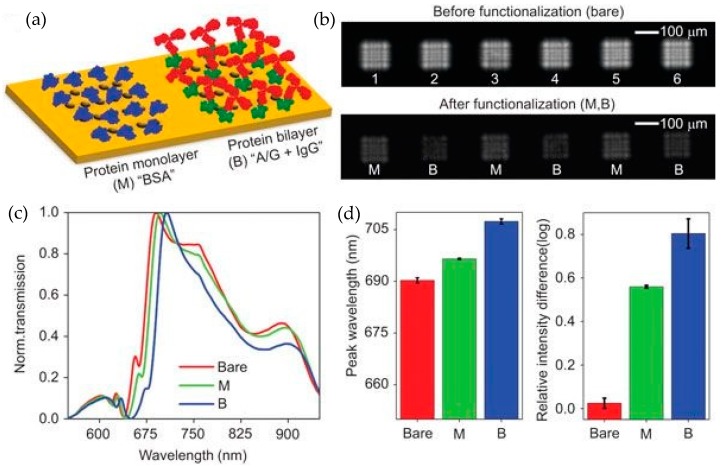
Experimental results detailed in Cetin et al. [77]. (**a**) Schematic of the performed protein assay. (**b**) Recorded lens free diffraction patterns of the bare nanohole array (top) and when mono (0.5% v/v BSA) and bi layer protein (0.5 mg/mL protein A/G + 0.5 mg/mL protein IgG) were deposited on the metallic nanoholes array (bottom). (**c**) Wavelength shift when mono and bi layers of protein were deposited on the metallic nanohole array (measured by an OSA). (**d**) Peak wavelength shift estimated from the lens free diffraction image patterns by relating resonance wavelength shift measured by the OSA shown in (c) and relative intensity difference of the diffraction patterns when mono and bi layer proteins are deposited shown in (b). Reprinted with permission from [77]. Copyright © CIOMP (2014).

To show the sensing performance of the developed sensor system, mono (M) and bi (B) layers of proteins were detected by recording diffraction image of the plasmonic nanostructures. The binding of 3 nm monolayer induced a 6 nm red shift of the resonance wavelength while an 11 nm bi layer gave a 17 nm shift as measured by the spectrum analyzer. As the LED emission was matched with the resonance response of the plasmonic nanoholes array, the diffraction pattern from unfunctionalized plasmonic array was bright, as the maximum intensity was recorded at resonance wavelength. The greater the resonance shift due to deposition of the protein layer the darker the diffraction pattern gets, as shown in Figure 13b, where the diffraction pattern from bi layer is darker compared to mono layer which in turn is darker than the unfunctionalized array. The quantification of the protein concentration was carried out by comparing the relative intensity change of the diffraction patterns with the optical resonance shift measured by an OSA. The optical sensitivity of the system was measured to be 621 nm/RIU, while the minimum relative intensity of the diffraction pattern was estimated as 0.024 which corresponds to a 3 nm wavelength shift for the analyte concentration in the order of µg/mL. The demonstrated sensor system has the potential of improvement in the detection limit by using a narrower linewidth incident light source and a higher performance CMOS imager. This work uses a smart technique to record the molecular binding instances. However, the quantification of the measured species still requires the comparison with the optical wavelength shift and therefore is still dependent on using external bulky equipment.

#### 3.2.2. Self-Readout of Nanophotonic Sensor Based on Chirped Gratings and Camera

To develop a portable nanophotonic sensor, Triggs et al., took a novel route of utilizing chirped resonant gratings [78]. The chirped resonant gratings were designed in such a way that they not only gave the resonance response but also translated the generated resonance signal into spatial information, which was read out by simple optical elements such as lens and a CCD camera.

**Figure 14:**
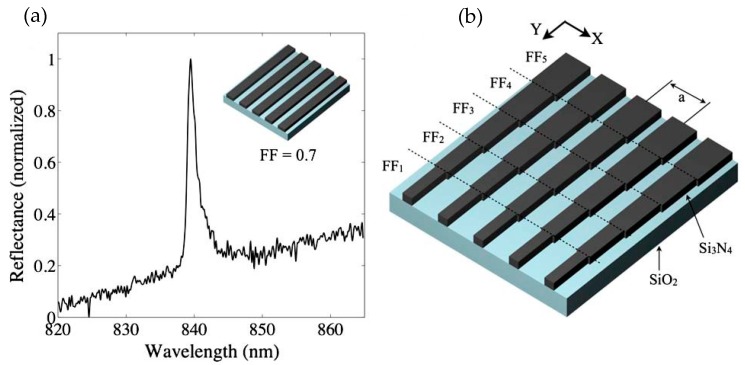
(**a**) Reflectance spectrum of standard rectangular gratings. (**b**) Schematic of chirped gratings. To induce chirping, the fill factor was changed along the Y direction which created a set of rectangular strips each having different fill factors along the Y-direction. Reprinted from [78] under CC BY 4.0.

On illuminating the gratings with polarized and collimated light from the top, a guided mode resonance was excited which gave rise to a reflectance/transmission peak at the resonance wavelength of the designed structures. Similar to conventional gratings or resonant structures, the change in the refractive index of the surrounding owing to molecular binding events induced a shift in the resonance peak. However, in this work instead of using an external spectrometer, the change in the resonance was measured by chirped gratings itself by making the resonance wavelength a function of the excitation area of the gratings. The gratings were chirped by tuning the fill factor (FF) of the gratings. The systematic tuning of the FF was performed by varying the dose of the e-beam exposure during fabrication. The sensor was composed of a set of grating strips each having dimensions of 6 µm × 500 µm. The chirped resonant gratings were designed and fabricated on a 150 nm thick silicon nitride layer on a glass slide and had a period and fill factor of 560 nm and 0.7 respectively. The schematic of the chirped grating sensor is shown in Figure 14.

The estimation of the change in the refractive index involved post processing of the measured reflection image of the grating. On illuminating the chirped grating array with monochromatic light, only a small part of the horizontal grating strip resonates and thus reflection occurs from only that localized region, whose intensity was recorded by the CCD camera.

**Figure 15:**
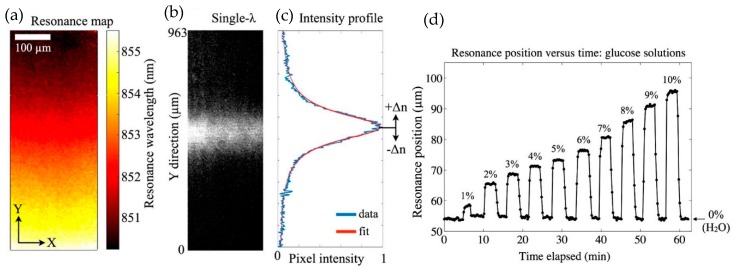
Experimental results presented in Triggs et al. [78]. (**a**) Resonance wavelength map when water is surrounding the gratings. (**b**) Measured brightfield image of the gratings when excited by a monochromatic light source. (**c**) Intensity profile of the measured resonance brightfield image shown in (**b**). The intensity profile is averaged and normalized. The change in refractive index of the grating cladding induces shift in the peak position of the intensity profile. (**d**) Shift in the peak position when the refractive index is changed by introducing different concentrations of glycerol. Water was added after each concentration measurement, which moved the peak position back to the original value (0% glycerol). Reprinted from [78] under CC BY 4.0.

The resonance position was then extracted by averaging and curve fitting to the measured intensity profile. A matlab code was used to perform the averaging and fitting operations. Any change in the refractive index due to binding events at the grating was quantified by using only a camera to measure the reflection intensity, a simple monochromatic light source to excite the gratings and image post-processing algorithm. The reflection image from the gratings immersed in water and extraction of the wavelength position by curve fitting is shown in Figure 15.

Since, this device works on measuring the spatial shift, the sensitivity value obtained by the sensor system was quoted in terms of position shift/RIU rather than the resonance wavelength shift/RIU. The sensitivity value in terms of position change per/RIU was reported to be 3469 µm/RIU, while the limit of detection value was reported as 2.37 × 10^−4^. 

**Figure 16:**
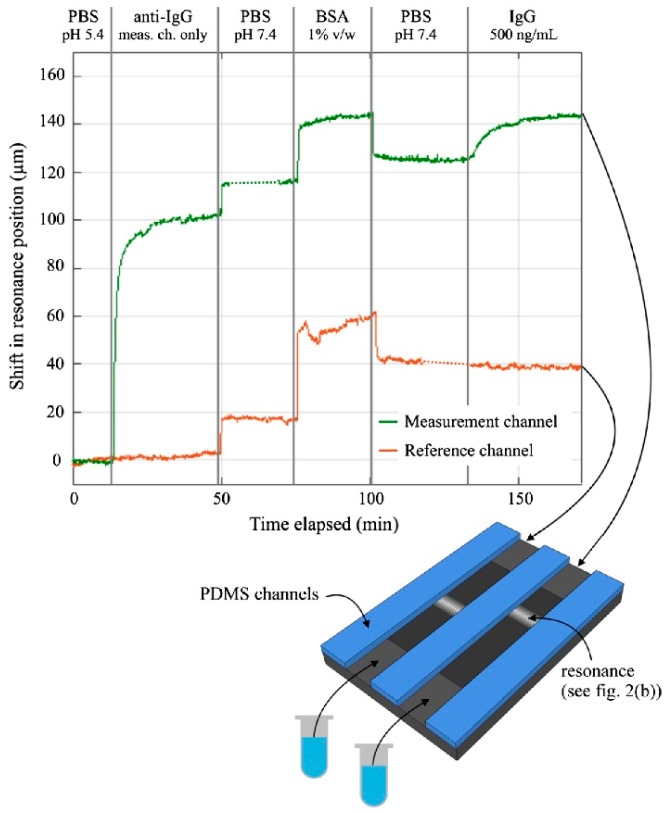
Schematic of the microfluidic channels integrated with the guided mode resonance (GMR) chirped grating sensor to apply the analyte as presented in Triggs et al. [78]. Two channels were formed which allowed introducing the analyte in one channel while second channel acted as a reference. The graph shows the measurement results (shift in the resonance position versus time) for the IgG assay performed on the GMR chirped grating sensors. Curves for both measurement (upper curve) and the reference (lower curve) channels are given with each assay step indicated on the top of the graph. Reprinted from [78] under CC BY 4.0.

Microfluidic channels were fabricated to perform the IgG binding assay. Two microfluidic channels were created, one for the measurements and the other for the reference. The shift in the resonance position as a function of time for both measurement and reference channels is shown in Figure 16. From the experimental results, the detection limit for the IgG assay was estimated to be 40 ng/mL. The estimated cost of the components used in this sensing system is relatively low at only U.S $10 and hence holds the potential of providing cheap portable sensors for point-of-care healthcare applications. 

## 4. Summary

The summary of the performance of the various onchip nanophotonic sensors having compact readout system as discussed in this article is given in Table 1.

## 5. Conclusions and Outlook

In conclusion, integrated nanophotonic structures have shown their potential for developing compact and high-performance sensors, i.e., which have high sensitivity and low detection limit, demonstrated by proof of concept laboratory based experiments. These sensors are made of different materials such as metals, semiconductors or dielectrics and generally require external bulky and expensive equipment for their measurement. The lack of compact measuring systems for onchip nanophotonic sensors has so far hindered their widespread application. In order, therefore, to transform nanophotonic sensors from a laboratory-based research topic to practical devices usable in everyday life, it is important to develop a compact and preferably integrated measurement system. Recognizing the importance of the need of a compact readout system, the efforts to develop a miniaturized measurement mechanism have been accelerated in the last few years with different approaches being followed. One approach is to monolithically integrate nanophotonic sensors with the photodetectors so that the resonance wavelength shift of the nanophotonic structures is read out as an electrical signal which can be interfaced with a portable gadget such as smart phone or tablet. Research that is following this approach has led to reports of integrating nanophotonic sensors with bulk silicon PDs, germanium PDs, and miniaturized PDs made by the CMOS technology in commercial foundries. Owing to the fact that CMOS technology has revolutionized the electronics and digital market, the monolithic integration of nanophotonic sensors with the PDs made by CMOS technology holds great promise for commercialization of nanophotonic sensors. In the reported work, the authors have integrated nanophotonic sensors with the CMOS PDs as a post processing in non-CMOS clean room; but the real commercialization opportunity lies in the complete fabrication of the sensor system in the CMOS commercial foundry. The large-scale production of the integrated sensor system in CMOS foundries will reduce the unit cost considerably. One major challenge to improving the performance of the CMOS integrated nanophotonic sensors is to reduce the back-reflection of the signal arising from different layers of the CMOS chip and to reduce the distance between the nanophotonic sensor and the underlying PD layer. Another approach that has been adopted by different groups to develop a compact readout mechanism for nanophotonic sensors is where a separate, yet small, imaging chip is used to read the output signal from the nanophotonic structures. Using an imaging chip such as a CMOS or CCD device to map the transmission response of the nanophotonic structures eliminates the need of external bulky and expensive laboratory-based equipment. However, as opposed to the monolithic integration approach, a housing is required to hold and align the nanophotonic sensor chip and the imaging chip and also a rigorous and computationally demanding post processing algorithm is necessary to obtain meaningful results. A summary of the performance of the various on-chip nanophotonic sensors that have a compact readout system is given in Table 1. The results presented in various works discussed in this article are highly dependent on the device used for the readout. For example, in the approach of monolithic integration of nanophotonic sensors with the PDs, the output signal depends highly on the responsivity and noise of the PD and the gain of the electronic readout circuitry. Similarly, in the approach, where separate CCD/CMOS imagers are used, the results also depend on the performance of the imager. In essence, both approaches have eliminated the need to use external and usually bulky equipment and therefore present a significant step towards developing practical nanophotonic sensors, enabling their application in everyday life. The work discussed in this review article has shown the sensing performance of several devices using basic analytes such as different concentrations of solutions or basic protein functionalization. We confidently expect that future design improvements to optimize the readout circuitry will improve performance of the sensor systems to such an extent that it will be possible to address clinically relevant analytes. By so doing, portable nanophotonic sensors will be used to target the diagnosis of many diseases including cancers and Alzheimer disease. 

## Figures and Tables

**Table 1:** Summary of onchip nanophotonic sensors having compact readout system.

References	Device	Integration	Analyte	Optical Sensitivity	Output of Integrated System	Detection Limit
Mazzotta et al. [68]	Gold nanodiscs integrated with bulk PD	Monolithic	Glycerol	133 nm/RIU	0.008 V change between Ref and analyte	0.01 RIU
Guyot et al. [69]	Gold nanohole array integrated with Si photodetector	Monolithic	Ethanol	N.A	0.8 V/RIU	1.75 × 10^−4^
Perino et al. [71]	Gold wire gratings integrated with Si photodetector	Monolithic	Dodecanethiol	N.A	2.94 a.u/ RIU	2.2 × 10^−4^
Turker et al. [72]	Silver coated polymer gratings	Monolithic	NaCl Solution	N.A	0.6 mA/RIU	
Augel et al. [73]	Aluminum hole array integrated with Ge PD	Monolithic	DI, water, IPA	N.A	1180 nm/RIU	N.A
Shakoor et al. [74,76]	Gold nanodiscs and silicon nitride gratings integrated with CMOS PD	Monolithic	Glycerol and protein	Gold nanodiscs: 270nm /RIU, Silicon nitride gratings: 160 nm/RIU	Gold nanodiscs: 5.6 V/RIU, Silicon nitride gratings: 6.75 V/RIU	3 × 10^−3^
Cetin et al. [77]	Gold nanohole array readout with CMOS imager	Not monolithic	Protein	621 nm/RIU	0.024 relative intensity change of diffraction pattern	1 µg/mL
Triggs et al. [78]	Chirped resonance gratings read by camera	Not monolithic	Glycerol and IgG	137 nm/RIU	3469 µm/RIU spatial shift (not wavelength shift)	2.37 × 10^−4^, 40 ng/mL
